# Relationship of Muscle Apolipoprotein E Expression with Markers of Cellular Stress, Metabolism, and Blood Biomarkers in Cognitively Healthy and Impaired Older Adults

**DOI:** 10.3233/JAD-221192

**Published:** 2023-04-04

**Authors:** Chelsea N. Johnson, Colin S. McCoin, Paul J. Kueck, Amelia G. Hawley, Casey S. John, John P. Thyfault, Russell H. Swerdlow, Paige C. Geiger, Jill K. Morris

**Affiliations:** a Department of Cell Biology and Physiology, University of Kansas Medical Center, Kansas City, KS, USA; b Department of Neurology, University of Kansas Medical Center, Kansas City, KS, USA; c Kansas University Alzheimer’s Disease Center, University of Kansas Medical Center, Kansas City, KS, USA; d Kansas University Diabetes Institute, University of Kansas Medical Center, Kansas City, KS, USA

**Keywords:** Alzheimer’s disease, *APOE4*, Hsp72, mitochondria, skeletal muscle, stress

## Abstract

**Background:**

Individuals with mild cognitive impairment (MCI) have reduced lipid-stimulated mitochondrial respiration in skeletal muscle. A major risk factor for Alzheimer’s disease (AD), the apolipoprotein E4 (*APOE4*) allele, is implicated in lipid metabolism and is associated with metabolic and oxidative stress that can result from dysfunctional mitochondria. Heat shock protein 72 (Hsp72) is protective against these stressors and is elevated in the AD brain.

**Objective:**

Our goal was to characterize skeletal muscle ApoE and Hsp72 protein expression in *APOE4* carriers in relationship to cognitive status, muscle mitochondrial respiration and AD biomarkers.

**Methods:**

We analyzed previously collected skeletal muscle tissue from 24 *APOE4* carriers (60y+) who were cognitively healthy (CH, *n* = 9) or MCI (*n* = 15). We measured ApoE and Hsp72 protein levels in muscle and phosphorylated tau181 (pTau181) levels in plasma, and leveraged previously collected data on *APOE* genotype, mitochondrial respiration during lipid oxidation, and VO_2_ max.

**Results:**

Muscle ApoE (*p* = 0.013) and plasma pTau181 levels (*p* < 0.001) were higher in MCI *APOE4* carriers. Muscle ApoE positively correlated with plasma pTau181 in all *APOE4* carriers (R^2^ = 0.338, *p* = 0.003). Hsp72 expression negatively correlated with ADP (R^2^ = 0.775, *p* = <0.001) and succinate-stimulated respiration (R^2^ = 0.405, *p* = 0.003) in skeletal muscle of MCI *APOE4* carriers. Plasma pTau181 negatively tracked with VO_2_ max in all *APOE4* carriers (R^2^ = 0.389, *p* = 0.003). Analyses were controlled for age.

**Conclusion:**

This work supports a relationship between cellular stress in skeletal muscle and cognitive status in *APOE4* carriers.

## INTRODUCTION

Approximately 16% of Americans age 65 years and older have mild cognitive impairment (MCI), and it is estimated that half of these cases are due to Alzheimer’s disease (AD), the most common form of dementia [1]. Impaired energy metabolism in the brain, characterized by reduced cerebral glucose metabolism [2], is a hallmark pathologic feature of AD. Metabolic dysfunction also manifests systemically in individuals with AD as reduced cardiorespiratory fitness [3], hypertriglyceridemia [4] and hyperglycemia [4].

Apolipoprotein E (ApoE) is a lipid transport protein, with various alleles modifying AD risk. When compared to non-carriers, *APOE4* carriers display more pronounced impairments in glucose meta-bolism in various brain regions [5], are more susceptible to metabolic syndrome [6], and are less responsive to interventions that target energy metabolism to improve cognitive function [7, 8]. Evidence that *APOE4* carrier status differentially affects both central and peripheral metabolism suggests that the underlying pathogenesis of AD varies by genotype and that whole-body metabolism may be more important to AD pathogenesis in *APOE4* carriers. These data support the notion that *APOE* genotype-specific effects on metabolism deserve further investigation.

Skeletal muscle mitochondrial function is critical for whole body oxygen consumption [9] and glucose and fatty acid oxidation [10]. In the absence of AD medication treatment, individuals with MCI have reduced mitochondrial respiration in skeletal muscle [11]. Data in preclinical rodent models further suggests that *APOE4* modifies skeletal muscle mitochondrial activity by increasing the expression of proteins implicated in fatty acid transport and oxidation [12].

ApoE and heat shock protein (Hsp72) are two proteins implicated in mitochondrial dysfunction and AD. Hsp72 is a highly-inducible, stress-responsive chaperone protein that has been found to be elevated in the temporal cortex of individuals with AD [13, 14]. In addition to its well-known roles in protein folding, this protein is responsive to and protective against declines in both ATP production [15] and oxidative stress [16, 17] that can result from mitochondrial dysfunction. Given that *APOE4* is associated with impairments in mitochondrial activity [18] and the ability to handle oxidative stress [19], Hsp72 may be critical to mitigating cellular stress in *APOE4* carriers.

Using skeletal muscle tissue collected from older adults who were cognitively healthy (CH) or MCI and at a genetic risk for AD (*APOE4* carriers), we investigated the hypothesis that ApoE and Hsp72 expression are elevated in skeletal muscle of *APOE4* carriers with MCI compared to those who are CH. We also determined if the expression of these proteins are inversely correlated with lipid-stimulated muscle mitochondrial respiration and positively correlated with plasma AD neuropathology biomarkers. We show that muscle ApoE and the plasma AD biomarker, phosphorylated tau181 (pTau181), are elevated in individuals with MCI and that plasma pTau181 is positively correlated with muscle ApoE and negatively correlated with VO_2_ max.

## MATERIALS AND METHODS

### Skeletal muscle and plasma

We leveraged a subset of vastus lateralis biopsy tissue and blood plasma banked from a previously completed study approved by the University of Kansas Medical Center’s Institutional Review Board (IRB #140787) [11]. Written informed consent was obtained from all participants. We analyzed tissue collected from a total of 24 *APOE4* carriers who were either cognitively healthy (*n* = 9) or were diagnosed with MCI (*n* = 15). Diagnostic inclusion criteria included no prior memory complaints (CH older adults), or MCI diagnosed by a clinician and verified with medical records. In the CH group, there were 7 E3/E4 and 2 E4/E4 participants. In the MCI group, there were 11 E3/E4 and 4 E4/E4 participants. All participants were 60 years or older.

### Western blot

Western blot analysis was performed on muscle tissue from CH (*n* = 8) and MCI (*n* = 15) *APOE4* carriers. One CH participant had insufficient tissue available for western blot. Frozen muscle was powdered and lysed with a TissueLyser II bead homogenizer in buffer (50 mM HEPES, 12 mM sodium pyrophosphate, 100 mM NaF, 10 mM EDTA, 400μl each of phosphatase inhibitor cocktails 1 and 2, and 1% Triton X-100). Protein concentration was measured using a BCA protein assay. Proteins were separated using SDS-PAGE prior to transferring proteins to a PVDF membrane. Proteins were stained with antibodies directed against ApoE (Abcam, Cambridge, MA; ab52607) and Hsp72 (Enzo Life Sciences, Farmingdale, NY; C92F3A-5) and protein loading was corrected using Ponceau S Solution (Abcam, Cambridge, MA; ab270042). Densitometry was used to quantify protein bands and total protein using Image Lab software (Bio-Rad Laboratories, Hercules, CA).

### Mitochondrial respiration and cardiorespiratory fitness

We leveraged mitochondrial O_2_ flux data and cardiorespiratory fitness data that was collected previously [11]. Briefly, vastus lateralis skeletal muscle tissue obtained at the time of biopsy was dissected to remove connective tissue and ∼30 mg was placed into ice cold buffer X as previously described [11]. Muscle fiber bundles were teased from the 30 mg of muscle and permeabilized for 30 min in 30μg/mL of saponin prior to being washed in buffer Z (105 mM K-MES, 30 mM KCl, 10 mM K2HPO4, 5 mM MgCl2-6H2O, and 0.5% w/w fatty acid-free BSA; pH 7.1) plus 0.5 M EGTA. O_2_ consumption rates in the presence of 4 mM ADP to assess state 3 respiration through complex I and 10 mM succinate to assess state 3 S respiration through complex II were measured in the presence of 0.018 mM palmitoylcarnitine (lipid), 0.01 mM blebbistatin, 0.02 mM palmitoyl CoA, 0.5 mM malate, and 5 mM carnitine in permeabilized muscle fiber bundles utilizing Buffer Z (plus 0.5M EGTA and 20 mM creatine monohydrate) on an Oroboros Oxygraph-2k system (Innsbruck, Austria) within 2-3 h of biopsy. Mitochondrial respiration values were normalized to dry muscle weight (pmol/s/mg dry weight per mL).

To assess cardiorespiratory fitness, a graded exercise test (GXT) was performed using a modified Bruce protocol [20]. Participants began by walking on a treadmill while speed and incline were gradually increased every 2 min as previously described [21]. Before and during the test, Respiratory Exchange Ratio (RER) was assessed using a non-rebreathing mask to capture oxygen and carbon dioxide levels. Cardiac rhythm was continuously monitored using a 12-lead electrocardiograph and at the end of each 2-min stage, blood pressure and rating of perceived exertion (RPE) were obtained. The test ended when participants reached volitional fatigue or met the absolute test termination criteria (RER> = 1.1, RPE 17, plateau in VO_2_ defined as 150 mL change from the last 60 s of the previous stage, and 90% HRmax). Maximal oxygen uptake was calculated relative to whole body mass (mL/kg/min). For the “VO_2_ max” analyses, we included individuals who met criteria for a “maximum” test (3/4 absolute test termination criteria met rather than just volitional fatigue) per ACSM guidelines [22].

### Plasma biomarkers

Phlebotomy was performed at the KU Clinical and Translational Science Unit following an overnight fast. Whole blood was stored for *APOE* genotyping and further processed to generate plasma as previously described [11]. For this study, pTau181 was measured in plasma on a Simoa HD-X (Quanterix, Billerica, MA) according to the manufacturer’s instructions. Data on amyloid-β 42 (Aβ_42_), amyloid-β 40 (Aβ_40_), neurofilament light (NFL), and glial fibrillary acidic protein (GFAP) measured previously on the Simoa HD-X using the Neurology 4-Plex E kit were also statistically analyzed in relation to our new outcomes of interest [11].

### Statistics

Mean group differences for continuous variables were assessed using ANOVA. Differences in nominal variables were assessed using a Chi-Square test. Linear associations between continuous variables were assessed using Pearson Correlation. All analyses were adjusted for age. Results were considered significant at *p*≤0.05.

## RESULTS

### Demographics

Individuals did not significantly differ based on demographic characteristics such as age, sex, education, or body mass index (Supplementary Table 1). Within the MCI group, the mean Mini-Mental State Examination score was 25 and 7 (46.7%) participants were taking the AD medication donepezil. There was no significant difference in lean mass between diagnostic groups.

**Fig. 1:**
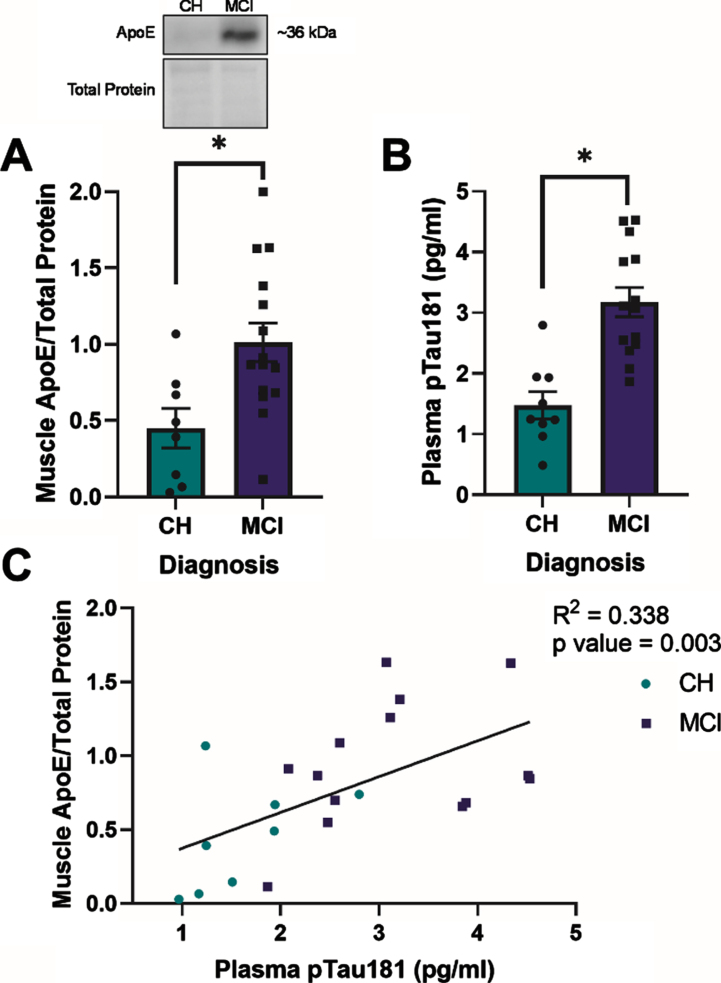
Apolipoprotein E (ApoE) expression in skeletal muscle and plasma phosphorylated tau181 (pTau181) expression are elevated in apolipoprotein ɛ4 (*APOE4)* carriers with mild cognitive impairment (MCI) and are positively correlated in all *APOE4* carriers. Mean muscle ApoE±standard error measured by Western blot in relationship to diagnostic status (A). Plasma pTau181±standard error measured by Simoa-HDX immunoassay in relationship to diagnostic status (B). ApoE muscle content in relationship to plasma pTau181 levels (C). CH, cognitively healthy older adults; MCI, mild cognitive impairment. CH *APOE4* carriers (*n* = 8-9), MCI *APOE4* carriers (*n* = 14-15). ^*^p < 0.001.

### Apolipoprotein E expression in skeletal muscle and relationship with AD neuropathology blood biomarkers

ApoE expression in skeletal muscle (*p* = 0.013) and plasma pTau181 expression (*p* < 0.001) were higher in *APOE4* carriers with MCI compared to those who were CH (Fig. 1A, B). In all *APOE4* carriers, there was a positive correlation between muscle ApoE protein content and plasma pTau181 levels (R^2^ = 0.338, *p* = 0.003) (Fig. 1C). Muscle ApoE content also correlated with plasma GFAP (R^2^ = 0.175, *p* = 0.007) and plasma NFL (R^2^ = 0.270, *p* = 0.009) levels in all participants (Supplementary Figure 1A, B). There was no relationship between muscle ApoE content and plasma Aβ_42 :40_ (*p* > 0.05, Supplementary Figure 1C). Although we observed diagnostic differences in ApoE protein levels between groups, ApoE protein content did not correlate with lipid-stimulated mitochondrial respiration in the presence of ADP or succinate in either group (*p* > 0.05, data not shown).

**Fig. 2:**
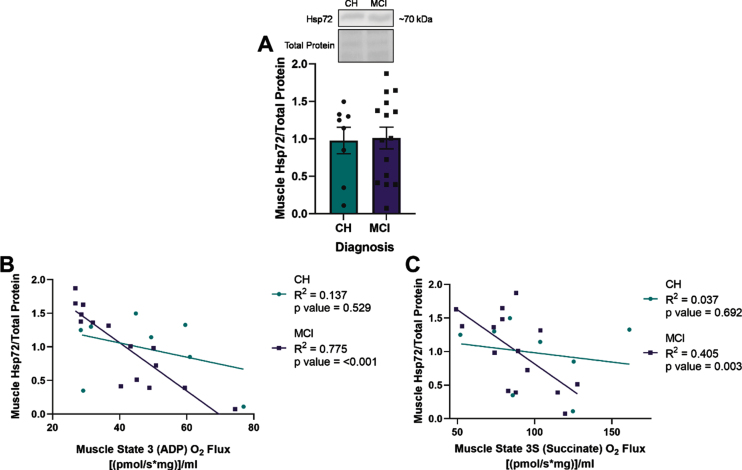
Heat shock protein 72 (Hsp72) expression negatively correlates with lipid-stimulated mitochondrial respiration in skeletal muscle of apolipoprotein ɛ4 (*APOE4)* carriers with mild cognitive impairment (MCI). Mean muscle Hsp72±standard error measured by Western blot in relationship to diagnostic status (A). Muscle Hsp72 in relationship to muscle State 3 (ADP) O_2_ flux (B) or State 3S (Succinate) O_2_ flux (C) measured by Oroboros. CH, cognitively healthy older adults; MCI, mild cognitive impairment. CH *APOE4* carriers (*n* = 8), MCI *APOE4* carriers (*n* = 15).

### Heat shock response and relationship with mitochondrial respiration in skeletal muscle

ADP- and succinate-stimulated mitochondrial respiration were non-significantly reduced in *APOE4* carriers with MCI compared to those who were CH (data not shown). There was no significant difference in muscle Hsp72 expression between diagnostic groups (Fig. 2A). However, we observed that Hsp72 expression negatively correlated with ADP (R^2^ = 0.775, *p* = <0.001) and succinate (R^2^ = 0.405, *p* = 0.003) stimulated mitochondrial respiration in the presence of palmitoyl-carnitine in skeletal muscle of *APOE4* carriers with MCI, but not in those were CH (Fig. 2A, B). Hsp72 did not correlate with plasma AD neuropathology biomarkers (*p* > 0.05, Supplementary Figure 2).

### AD neuropathology blood biomarkers and relationship with fitness

**Fig. 3:**
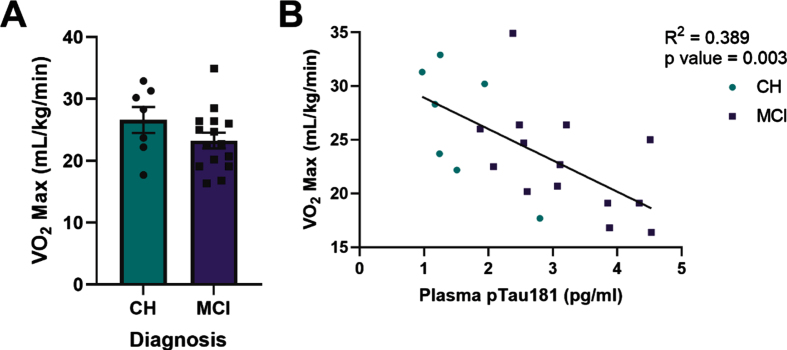
Plasma phosphorylated tau181 (pTau181) negatively correlates with VO_2_ max in all apolipoprotein ɛ4 (*APOE4)* carriers. VO_2_ max in relationship to diagnostic status (A). Plasma pTau181 measured by Simoa-HDX immunoassay in relationship to VO_2_ max in all *APOE4* carriers (B). CH, cognitively healthy older adults; MCI, mild cognitive impairment. CH *APOE4* carriers (*n* = 7), MCI *APOE4* carriers (*n* = 14-15).

Cardiorespiratory fitness (VO_2_ max) did not significantly differ by diagnostic group in *APOE4* carriers (Fig. 3A). However, plasma pTau181 (R^2^ = 0.389, *p* = 0.003, Fig. 3B) and GFAP (Supplementary Table 2) negatively correlated with VO_2_ max in all *APOE4* carriers. Within diagnostic groups, plasma GFAP and NFL negatively correlated with VO_2_ max in *APOE4* carriers who were cognitively healthy, but not in those with MCI (Supplementary Table 2). There was no relationship between Aβ_42 :40_ ratio and VO_2_ max.

## DISCUSSION

Research over the past few decades supports the notion that AD is a systemic disease [23]. Evidence that skeletal muscle mitochondrial respiration is reduced in the early stages of AD-related cognitive decline [11] extends previous studies that show mitochondrial and metabolic dysfunction in AD platelets and fibroblasts [24], and suggests that systemic metabolic tissues may influence or reflect AD progression. Skeletal muscle comprises 40–50% of total body mass and is a critical component for maintenance of physical function and metabolism of aging. In this study, we show for the first time that muscle ApoE levels are increased in *APOE4* carriers with MCI compared to those who are CH and that Hsp72 levels negatively correlate with mitochondrial respiration in the muscle of *APOE4* carriers with MCI, but not in those who are CH. We further show that the plasma AD biomarker, pTau181 negatively tracks with cardiorespiratory fitness in all *APOE4* carriers. By demonstrating skeletal muscle changes in proteins implicated in responding to metabolic and oxidative stress in *APOE4* carriers, we contribute novel information to the existing literature on systemic alterations in AD.

Recent animal studies have focused on the role of *APOE4* in modulating systemic metabolism [12, 25, 26]. In addition to impaired spatial learning [25], mice expressing human *APOE4* have impaired glucose clearance at lower body weights [25], increased plasma insulin [26], elevated levels of fasting plasma non-esterified fatty acids [12], and altered skeletal muscle expression of proteins related to mitochondrial energy metabolism [12]. Similarly, human *APOE4* carriers have higher plasma triglyceride levels in the fasted and post-prandial state in addition to elevated post-prandial glucose [27]. Although the role of ApoE4 protein levels in modulating systemic metabolic changes is unclear, human *APOE4* carriers have altered plasma levels of ApoE compared to non-carriers [28]. Further, the expression of ApoE4 fragments in Neuro-2a cells reduces the expression of Mitotracker red, a marker of mitochondrial function and integrity, in a dose-dependent manner [18]. We therefore were not surprised to find differences in ApoE protein levels in MCI compared to CH individuals [11].

As mentioned, muscle ApoE protein is elevated in *APOE4* carriers with MCI compared to those who are CH. While increased levels of muscle ApoE may still contribute to reduced muscle mitochondrial respiration observed in MCI, ApoE protein levels did not correlate with ADP-stimulated or succinate-stimulated mitochondrial respiration utilizing lipids as a substrate in either group. This lack of relationship may be due to our limited sample size, medication use [11], or other factors. Preclinical models have shown that *APOE4* alters the mobilization and utilization of fatty acids by adipose and skeletal muscle tissue [12, 26]. *APOE4* transgenic mice preferentially mobilize fats from adipose tissue and increase the expression of proteins needed to oxidize fats in skeletal muscle [12]. Excess flux of lipids into muscle mitochondria and generation of reactive oxygen species may therefore be another mechanism by which mitochondrial respiration is impaired in MCI. *APOE4* is associated with excess oxidative stress [29], which is handled by cells through various mechanisms, including heat shock proteins. Hsp72 may mitigate reductions in mitochondrial function in some MCI individuals who have elevated expression of ApoE in skeletal muscle.

Hsp72 is a chaperone protein whose expression is activated in response to cellular insults including misfolded proteins, oxidative stress, and reduced ATP availability [15, 16]. Hsp72 protein is elevated in the temporal cortex of individuals with AD [13, 14], suggesting activation of this system in response to AD-related cellular stress. While no studies have measured muscle Hsp72 content in skeletal muscle of individuals with AD, overexpression of Hsp72 in mouse skeletal muscle improves whole-body VO_2_, muscle mitochondrial-content and -respiration [30], while mice lacking Hsp72 display reduced oxygen consumption and fatty acid oxidation in muscle [31]. Since this protein is activated in the AD brain and because mitochondrial dysfunction can stimulate the heat shock response [15, 17], we hypothesized that Hsp72 would be elevated in MCI muscle compared to CH. The lack of difference in Hsp72 muscle levels between CH and MCI *APOE4* carriers may be due to confounding effects of AD medications [11] or the availability of compensatory heat shock proteins [16] to deal with mitochondrial stress in some individuals. Detection of group differences in the expression of Hsp72 may also be limited by a state of chronic adaptation and compensation in these human samples. Compensatory mechanisms may also explain the absence of a statistically significant correlation between muscle Hsp72 and plasma AD biomarkers. Despite the lack of between-group differences in Hsp72 levels, the negative correlation between Hsp72 and lipid-stimulated mitochondrial respiration in skeletal muscle of *APOE4* carriers with MCI supports a role for Hsp72 in responding to mitochondrial dysfunction during cognitive decline. In a cognitively healthy state, there may be compensatory stress-responsive mechanisms other than Hsp72 available to deal with impaired mitochondrial respiration. In contrast, MCI may represent a state of increased reliance on the Hsp72 chaperone system to respond to mitochondrial stress due to a failure of other pathways. Further studies are needed to determine if systemic activation of Hsp72 in *APOE4* carriers with low mitochondrial respiration and MCI is protective against declines in mitochondrial activity and if these changes are representative of or influence changes in the brain.

Increasing expression of Hsp72 in response to declining mitochondrial respiration in skeletal muscle of *APOE4* carriers with MCI may also represent increased oxidative stress, since poorly functioning mitochondria produce excess reactive oxygen species that can damage a cell by oxidizing membrane lipids, proteins, and DNA [32]. We therefore hypothesized that proteins associated with metabolic/oxidative stress in muscle would be related to metabolic/oxidative stress related pathology in the brain. Oxidative stress increases the phosphorylation of tau [32], although the extent to which metabolic and oxidative stress in peripheral tissues reflects tau phosphorylation in the brain is not clear. We hypothesized that plasma pTau181, a biomarker for tau hyperphosphorylation in the brain, would track with Hsp72 expression in skeletal muscle of *APOE4* carriers. As expected, plasma pTau181 expression was higher in MCI compared to CH *APOE4* carriers, consistent with data showing that tau accumulation tracks with cognitive decline [33]. It is also known that ApoE4 enhances tau phosphorylation [34]. Although muscle Hsp72 did not correlate with plasma pTau181 in *APOE4* carriers, the negative correlation between plasma pTau181 levels and VO_2_ max in all *APOE4* carriers supports a potential connection between brain neuropathology and whole-body metabolism. The importance of Hsp72 localization [35] may underlie the lack of association between whole-muscle cell Hsp72 measured in our study and plasma pTau181.

Although the interpretation of our results is limited by its cross-sectional design and small sample size, the strengths of this study should be noted. This study is one of few to utilize human skeletal muscle tissue to assess differences related to cognitive status in systemic tissues. This data is paired with rich AD biomarker data that reflects expected differences between diagnostic groups. Furthermore, we demonstrate diagnostic dependent relationships in a highly metabolic and abundant tissue that can be used to help further our understanding of how whole-body metabolism is related to cognitive function in *APOE4* carriers. As this was an exploratory study, we were limited by the existing sample size. Future studies are needed to confirm these results and determine the implications of these findings.

The effects of *APOE4* are not limited to the brain. *APOE4* increases the risk of metabolic syndrome [36] and diseases characterized by peripheral metabolic dysfunction such as obesity and type 2 diabetes, are also risk factors for AD [37]. Therefore, understanding the systemic metabolic effects of *APOE4* is crucial to elucidating the mechanisms by which carrying an *APOE4* allele contributes to cognitive decline. Our work reveals diagnostic dependent differences in skeletal muscle between individuals who are CH or have been diagnosed with MCI but both carry the *APOE4* genotype. This raises the potential for skeletal muscle to be used as an early, minimally invasive biomarker for neuropathologic and cognitive changes. Furthermore, our work adds to the growing body of literature demonstrating systemic changes in AD.

## Supplementary Material

Supplementary Material

## Data Availability

The dataset used in the current study has been uploaded to the Harvard Dataverse repository and will be made publicly available at https://doi.org/10.7910/DVN/VDBMPPfollowingpublication.

## References

[1] (2022) 2022 Alzheimer’s disease facts and figures. Alzheimers Dement 18, 700–789. 35289055 10.1002/alz.12638

[2] Neth BJ , Craft S (2017) Insulin resistance and Alzheimer’s disease: Bioenergetic linkages. Front Aging Neurosci 9, 345. 29163128 10.3389/fnagi.2017.00345 PMC5671587

[3] Burns JM , Cronk BB , Anderson HS , Donnelly JE , Thomas GP , Harsha A , Brooks WM , Swerdlow RH (2008) Cardiorespiratory fitness and brain atrophy in early Alzheimer disease. Neurology 71, 210–216. 18625967 10.1212/01.wnl.0000317094.86209.cb PMC2657657

[4] Razay G , Vreugdenhil A , Wilcock G (2007) The metabolic syndrome and Alzheimer disease. Arch Neurol 64, 93–96. 17210814 10.1001/archneur.64.1.93

[5] Mosconi L , Nacmias B , Sorbi S , De Cristofaro MT , Fayazz M , Tedde A , Bracco L , Herholz K , Pupi A (2004) Brain metabolic decreases related to the dose of the ApoE e4 allele in Alzheimer’s disease. J Neurol Neurosurg Psychiatry 75, 370–376. 14966149 10.1136/jnnp.2003.014993 PMC1738980

[6] Sima A , Iordan A , Stancu C (2007) Apolipoprotein E polymorphism–a risk factor for metabolic syndrome. Clin Chem Lab Med 45, 1149–1153. 17848120 10.1515/CCLM.2007.258

[7] Craft S , Asthana S , Schellenberg G , Baker L , Cherrier M , Boyt AA , Martins RN , Raskind M , Peskind E , Plymate S (2000) Insulin effects on glucose metabolism, memory, and plasma amyloid precursor protein in Alzheimer’s disease differ according to apolipoprotein-E genotype. Ann N Y Acad Sci 903, 222–228. 10818510 10.1111/j.1749-6632.2000.tb06371.x

[8] Huang TL , Zandi PP , Tucker KL , Fitzpatrick AL , Kuller LH , Fried LP , Burke GL , Carlson MC (2005) Benefits of fatty fish on dementia risk are stronger for those without APOE ɛ4. Neurology 65, 1409–1414. 16275829 10.1212/01.wnl.0000183148.34197.2e

[9] Ivy JL , Costill DL , Maxwell BD (1980) Skeletal muscle determinants of maximum aerobic power in man. Eur J Appl Physiol Occup Physiol 44, 1–8. 7190491 10.1007/BF00421757

[10] Merz KE , Thurmond DC (2020) Role of skeletal muscle in insulin resistance and glucose uptake. Compr Physiol 10, 785–809. 32940941 10.1002/cphy.c190029 PMC8074531

[11] Morris JK , McCoin CS , Fuller KN , John CS , Wilkins HM , Green ZD , Wang X , Sharma P , Burns JM , Vidoni ED , Mahnken JD , Shankar K , Swerdlow RH , Thyfault JP (2021) Mild cognitive impairment and donepezil impact mitochondrial respiratory capacity in skeletal muscle. Function (Oxf) 2, zqab045. 34661111 10.1093/function/zqab045 PMC8515006

[12] Huebbe P , Dose J , Schloesser A , Campbell G , Glüer CC , Gupta Y , Ibrahim S , Minihane AM , Baines JF , Nebel A , Rimbach G (2015) Apolipoprotein E (APOE) genotype regulates body weight and fatty acid utilization-Studies in gene-targeted replacement mice. Mol Nutr Food Res 59, 334–343. 25381750 10.1002/mnfr.201400636

[13] Koren J , 3rd, Jinwal UK , Lee DC , Jones JR , Shults CL , Johnson AG , Anderson LJ , Dickey CA (2009) Chaperone signalling complexes in Alzheimer’s disease. J Cell Mol Med 13, 619–630. 19449461 10.1111/j.1582-4934.2008.00557.x PMC2749087

[14] Yoo BC , Seidl R , Cairns N , Lubec G (1999) Heat-shock protein 70 levels in brain of patients with Down syndrome and Alzheimer’s disease. J Neural Transm Suppl 57, 315–322. 10666686 10.1007/978-3-7091-6380-1_22

[15] Van Why SK , Mann AS , Thulin G , Zhu XH , Kashgarian M , Siegel NJ (1994) Activation of heat-shock transcription factor by graded reductions in renal ATP, *in vivo*, in the rat. J Clin Invest 94, 1518–1523. 7929828 10.1172/JCI117492 PMC295298

[16] Kregel KC (2002) Heat shock proteins: Modifying factors in physiological stress responses and acquired thermotolerance. J Appl Physiol (1985) 92, 2177–2186. 11960972 10.1152/japplphysiol.01267.2001

[17] Barrett MJ , Alones V , Wang KX , Phan L , Swerdlow RH (2004) Mitochondria-derived oxidative stress induces a heat shock protein response. J Neurosci Res 78, 420–429. 15389841 10.1002/jnr.20249

[18] Chang S , ran Ma T , Miranda RD , Balestra ME , Mahley RW , Huang Y (2005) Lipid- and receptor-binding regions of apolipoprotein E4 fragments act in concert to cause mitochondrial dysfunction and neurotoxicity. Proc Natl Acad Sci U S A 102, 18694–18699. 16344479 10.1073/pnas.0508254102 PMC1311737

[19] Miyata M , Smith JD (1996) Apolipoprotein E allele-specific antioxidant activity and effects on cytotoxicity by oxidative insults and beta-amyloid peptides. Nat Genet 14, 55–61. 8782820 10.1038/ng0996-55

[20] Billinger SA , Vidoni ED , Greer CS , Graves RS , Mattlage AE , Burns JM (2014) Cardiopulmonary exercise testing is well tolerated in people with Alzheimer-related cognitive impairment. Arch Phys Med Rehabil 95, 1714–1718. 24780290 10.1016/j.apmr.2014.04.007 PMC4149924

[21] Morris JK , Vidoni ED , Johnson DK , Van Sciver A , Mahnken JD , Honea RA , Wilkins HM , Brooks WM , Billinger SA , Swerdlow RH , Burns JM (2017) Aerobic exercise for Alzheimer’s disease: A randomized controlled pilot trial. PLoS One 12, e0170547. 28187125 10.1371/journal.pone.0170547 PMC5302785

[22] Liguori G (2022) ACSM’s Guidelines for Exercise Testing and Prescription, 11 ed. Wolters Kluwer Health.

[23] Morris JK , Honea RA , Vidoni ED , Swerdlow RH , Burns JM (2014) Is Alzheimer’s disease a systemic disease? Biochim Biophys Acta 1842, 1340–1349. 24747741 10.1016/j.bbadis.2014.04.012 PMC4126236

[24] Swerdlow RH (2012) Mitochondria and cell bioenergetics: Increasingly recognized components and a possible etiologic cause of Alzheimer’s disease. Antioxid Redox Signal 16, 1434–1455. 21902597 10.1089/ars.2011.4149 PMC3329949

[25] Jones NS , Watson KQ , Rebeck GW (2019) Metabolic disturbances of a high-fat diet are dependent on APOE genotype and sex. eNeuro 6, ENEURO.0267-19.2019. 10.1523/ENEURO.0267-19.2019 PMC6795556 31554665

[26] Arbones-Mainar JM , Johnson LA , Torres-Perez E , Garcia AE , Perez-Diaz S , Raber J , Maeda N (2016) Metabolic shifts toward fatty-acid usage and increased thermogenesis are associated with impaired adipogenesis in mice expressing human APOE4. Int J Obes (Lond) 40, 1574–1581. 27163745 10.1038/ijo.2016.93 PMC5063049

[27] Dart A , Sherrard B , Simpson H (1997) Influence of apo E phenotype on postprandial triglyceride and glucose responses in subjects with and without coronary heart disease. Atherosclerosis 130, 161–170. 9126660 10.1016/s0021-9150(96)06062-5

[28] Martínez-Morillo E , Hansson O , Atagi Y , Bu G , Minthon L , Diamandis EP , Nielsen HM (2014) Total apolipoprotein E levels andspecific isoform composition in cerebrospinal fluid and plasma fromAlzheimer’s disease patients and controls. Acta Neuropathol 127, 633–643. 24633805 10.1007/s00401-014-1266-2

[29] Orr AL , Kim C , Jimenez-Morales D , Newton BW , Johnson JR , Krogan NJ , Swaney DL , Mahley RW (2019) Neuronal apolipoprotein E4 expression results in proteome-wide alterations and compromises bioenergetic capacity by disrupting mitochondrial function. J Alzheimers Dis 68, 991–1011. 30883359 10.3233/JAD-181184 PMC6481541

[30] Henstridge DC , Bruce CR , Drew BG , Tory K , Kolonics A , Estevez E , Chung J , Watson N , Gardner T , Lee-Young RS , Connor T , Watt MJ , Carpenter K , Hargreaves M , McGee SL , Hevener AL , Febbraio MA (2014) Activating HSP72 in rodent skeletal muscle increases mitochondrial number and oxidative capacity and decreases insulin resistance. Diabetes 63, 1881–1894. 24430435 10.2337/db13-0967 PMC4030108

[31] Drew BG , Ribas V , Le JA , Henstridge DC , Phun J , Zhou Z , Soleymani T , Daraei P , Sitz D , Vergnes L , Wanagat J , Reue K , Febbraio MA , Hevener AL (2014) HSP72 is a mitochondrial stress sensor critical for Parkin action, oxidative metabolism, and insulin sensitivity in skeletal muscle. Diabetes 63, 1488–1505. 24379352 10.2337/db13-0665 PMC3994950

[32] Mondragón-Rodríguez S , Perry G , Zhu X , Moreira PI , Acevedo-Aquino MC , Williams S (2013) Phosphorylation of tau proteinas the link between oxidative stress, mitochondrial dysfunction, andconnectivity failure: Implications for Alzheimer’s disease. Oxid Med Cell Longev 2013, 940603. 23936615 10.1155/2013/940603 PMC3723250

[33] Gomes LC , Benedetto G , Scorrano L (2011) During autophagy mitochondria elongate, are spared from degradation and sustain cell viability. Nat Cell Biol 13, 589–598. 21478857 10.1038/ncb2220 PMC3088644

[34] Brecht WJ , Harris FM , Chang S , Tesseur I , Yu GQ , Xu Q , Dee Fish J , Wyss-Coray T , Buttini M , Mucke L , Mahley RW , Huang Y (2004) Neuron-specific apolipoprotein e4 proteolysis is associated with increased tau phosphorylation in brains of transgenic mice. J Neurosci 24, 2527–2534. 15014128 10.1523/JNEUROSCI.4315-03.2004 PMC6729489

[35] Ellis S , Killender M , Anderson RL (2000) Heat-induced alterations in the localization of HSP72 and HSP73 as measured by indirect immunohistochemistry and immunogold electron microscopy. J Histochem Cytochem 48, 321–332. 10681386 10.1177/002215540004800302

[36] Torres-Perez E , Ledesma M , Garcia-Sobreviela MP , Leon-Latre M , Arbones-Mainar JM (2016) Apolipoprotein E4 association with metabolic syndrome depends on body fatness. Atherosclerosis 245, 35–42. 26691908 10.1016/j.atherosclerosis.2015.11.029

[37] Profenno LA , Porsteinsson AP , Faraone SV (2010) Meta-analysis of Alzheimer’s disease risk with obesity, diabetes, and related disorders. Biol Psychiatry 67, 505–512. 19358976 10.1016/j.biopsych.2009.02.013

