# Supplementary material for: Relationship of Muscle Apolipoprotein E Expression with Markers of Cellular Stress, Metabolism, and Blood Biomarkers in Cognitively Healthy and Impaired Older Adults
Source: J Alzheimers Dis. 2023 Apr 4;92(3):1027–35. doi: 10.3233/JAD-221192 (PMC10116140; doi:10.3233/JAD-221192)
Supplement: Supplementary Material [file jad-92-jad221192-s001.pdf]

Supplementary Material

Relationship of Muscle Apolipoprotein E Expression with Markers of Cellular Stress, Metabolism, and Blood Biomarkers in Cognitively Healthy and Impaired Older Adults

Supplementary Table 1. Participant characteristics

	CH (n=9)	MCI (n=15)	p
Age (y)	73.5 [10.2]	72.2 [6.2]	0.734
Sex (#, % female)	4 (44%)	12 (80%)	0.074
Education (y)	16.7 [2.0]	16.5 [2.5]	0.893
BMI	28.8 [6.1]	25.2 [6.1]	0.177

CH, cognitively healthy; MCI, mild cognitive impairment; BMI, body mass index. Mean values are presented as mean [SD] and considered significant at $p<0.05$.

Supplementary Table 2. Pearson correlations between VO_2 Max and plasma Alzheimer's disease biomarkers in apolipoprotein ε4 (*APOE4)* carriers.

	All *APOE4* Carriers	CH *APOE4* Carriers	MCI *APOE4* Carriers
Plasma Biomarker		VO_2 Max	
pTau181	-0.624*	-0.712	-0.408
$A\beta_{42:40}$	0.362	0.701	0.296
GFAP	-0.782*	-0.932*	-0.538
NFL	-0.435	-0.912*	0.258

Values shown are Pearson Correlation Coefficients. CH, cognitively healthy; MCI, mild cognitive impairment; pTau181, phosphorylated tau181; $A\beta_{40}$, amyloid-beta 40; $A\beta_{42}$, amyloid-beta 42; GFAP, glial fibrillary acidic protein; NFL, neurofilament light. *$p<0.05$.

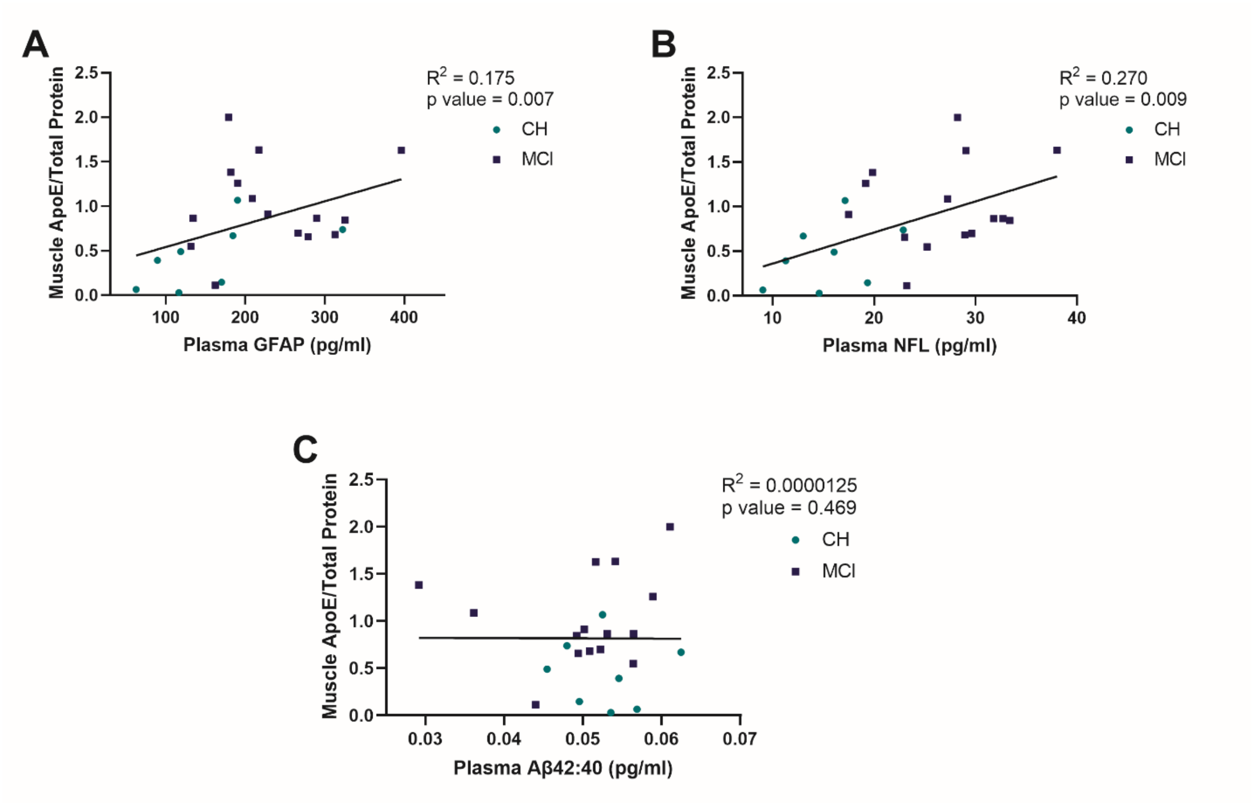

Supplementary Figure 1. Apolipoprotein E (ApoE) expression in relationship to plasma Alzheimer's disease (AD) biomarkers in apolipoprotein ε4 (*APOE4)* carriers. ApoE muscle content measured by western blot in relationship to plasma biomarkers measured by Simoa-HDX immunoassay, glial fibrillary acidic protein (GFAP) (A), neurofilament light (NFL) (B), and $A\beta_{42:40}$ (C). CH, cognitively healthy older adults; MCI, mild cognitive impairment. CH *APOE4* carriers (n=8), MCI *APOE4* carriers (n=15).

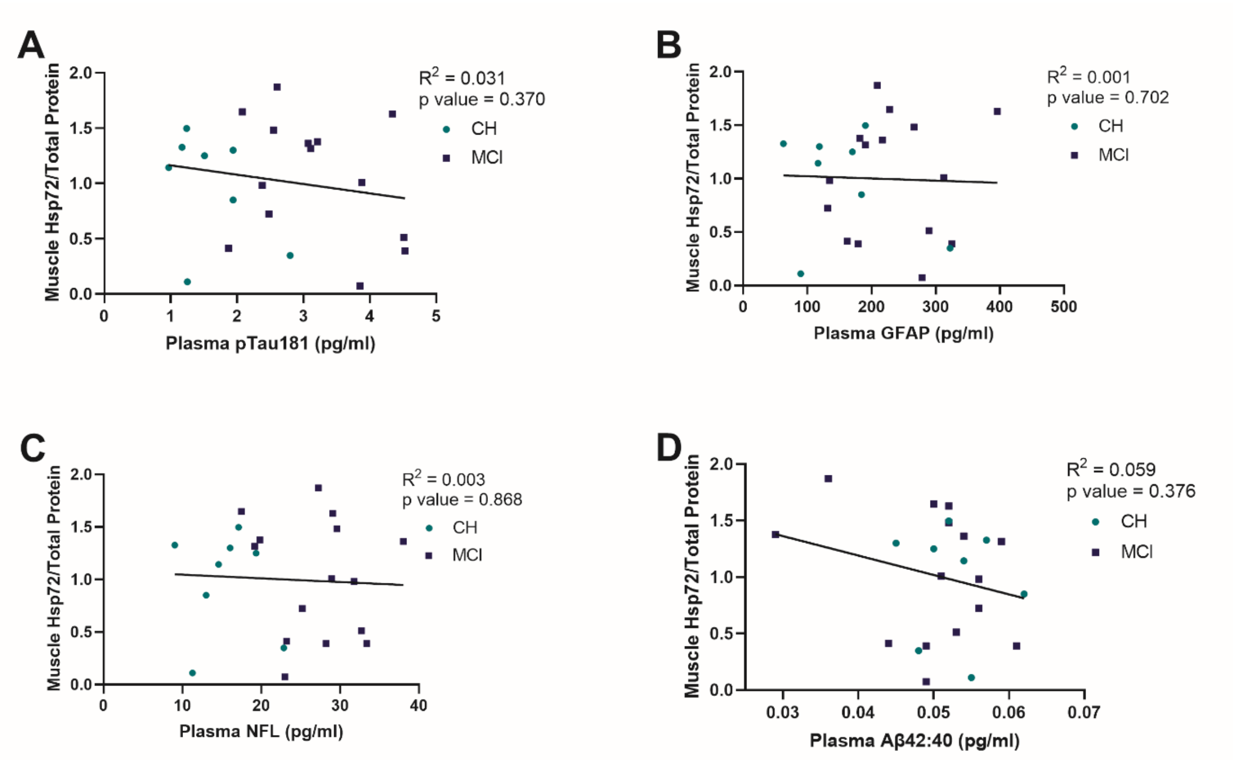

Supplementary Figure 2. Heat shock protein 72 (Hsp72) expression in relationship to plasma Alzheimer's disease (AD) biomarkers in apolipoprotein ε4 (*APOE4)* carriers. Hsp72 muscle content measured by western blot in relationship to plasma biomarkers measured by Simoa-HDX immunoassay, plasma pTau181 (pTau181) (A), glial fibrillary acidic protein (GFAP) (B), neurofilament light (NFL) (C), and $A\beta_{42:40}$ (D). CH, cognitively healthy older adults; MCI, mild cognitive impairment. CH *APOE4* carriers (n=8), MCI *APOE4* carriers (n=14-15).
